# Expression of CD82 in Human Trophoblast and Its Role in Trophoblast Invasion

**DOI:** 10.1371/journal.pone.0038487

**Published:** 2012-06-05

**Authors:** Qian Zhang, Dongmei Tan, Wenping Luo, Junjie Lu, Yi Tan

**Affiliations:** Laboratory Animal Center, Chongqing Medical University, Chongqing, China; VU University Medical Center, The Netherlands

## Abstract

**Background:**

Well-controlled trophoblast invasion at maternal-fetal interface is a critical event for the normal development of placenta. CD82 is a member of transmembrane 4 superfamily, which showed important role in inhibiting tumor cell invasion and migration. We surmised that CD82 are participates in trophoblast differentiation during placenta development.

**Methodology/Principal Findings:**

CD82 was found to be strongly expressed in human first trimester placental villous and extravillous trophoblast cells as well as in trophoblast cell lines. To investigate whether CD82 plays a role in trophoblast invasion and migration, we further utilized human villous explants culture model on matrigel and invasion/migration assay of trophoblast cell line HTR8/SVneo. CD82 siRNA significantly promoted outgrowth of villous explants *in vitro* (*P*<0.01), as well as invasion and migration of HTR8/SVneo cells (*P*<0.05), whereas the trophoblast proliferation was not affected. The enhanced effect of CD82 siRNA on invasion and migration of trophoblast cells was found associated with increased gelatinolytic activities of matrix metalloproteinase MMP9 while over-expression of CD82 markedly decreased trphoblast cell invasion and migration as well as MMP9 activities.

**Conclusions/Significance:**

These findings suggest that CD82 is an important negative regulator at maternal-fetal interface during early pregnancy, inhibiting human trophoblast invasion and migration.

## Introduction

The invasion of uterine decidua and maternal vasculature by trophoblast cells is a critical process for successful establishment the maternal-fetal circulation and therefore ongoing pregnancy. During human placental development, three primary types of trophoblast populations have been identified, cytotrophoblasts (CTBs), syncytiotrophoblast (STB) and extrovillous trophoblasts (EVTs), each has a specific role in regulating trophoblst invasion [1]–[3]. Trophoblast invasion is characterized by strict spatio-temporal regulation, which is mediated in an autocrine or paracrine manner by trophoblastic and uterine factors at the maternal-fetal interface. For example, EVTs secreting matrix metalloproteinases (MMPs) are invasive in nature, and play important role in cell-cell adhesion and migration [4]–[7], while tissue inhibitor of MMP (TIMP) inhibits their invasiveness. There is a complex network of cell types, mediators and signaling pathways regulating trophoblast invasiveness by different hormones, growth factors, cytokines and chemokines [8]–[15].

The metastasis suppressor gene CD82 (KAI1) is a member of the transmembrane 4 protein superfamily (TM4SF). The role of CD82 in inhibiting cancer progression was discovered during a genetic screen to identify metastasis suppressor genes [16]. CD82 can form complexes with integrins and other teteaspanins to regulate intercellular adhesion and transduction and the complexes induced intracellular signaling regulating cell proliferation, activation, and motility [17]–[20]. To date, the inhibitory role of CD82 in cancer progression has been well established and related molecular mechanisms have been intensively studied. Recently, it was reported that CD82 was expressed at the human maternal-fetal interface [21] mainly at the decidual compartment [22]–[24], while its expression and role in normal trophoblast cells is not clear. We noticed that two sets of microarray analysis revealed that CD82 was strongly expressed in the human placenta [25], [26]. There is a possibility that CD82 might also express in the trophoblast cell lineages. And we constantly detected CD82 transcription in several trophoblast cell lines; these clues raised the possibility that CD82 might also play an uncovered role in the trophoblastic lineages at the maternal-fetal interface. Based on this hypothesis, here we investigated the spatio-temporal expression of CD82 at the human maternal-fetal interface and its effects on trophoblast cell outgrowth/invasion by using villous explant culture model as well as trophoblast cell lines.

## Results

### CD82 Expressed in First Trimester Human Placental Villous and Invasive Extravillous Trophoblast

**Figure 1:**
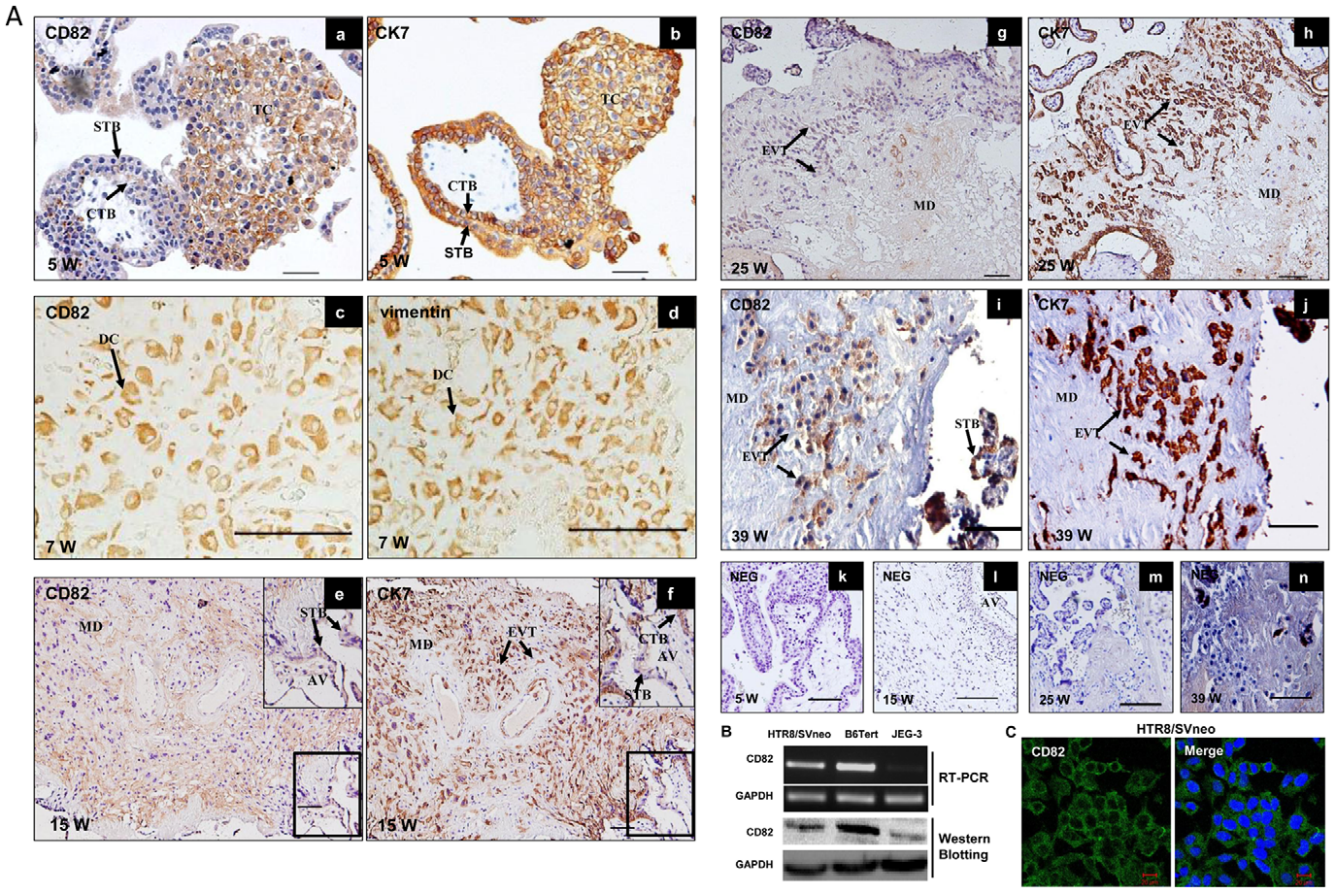
Expression of CD82 in human placental villi and cell lines. (**A**) Immunostaining of CD82 in normal human placental villi in the first trimester, maternal decidua, second trimester and third trimester. (a) CD82 was strongly expressed in trophoblast columns (TC) and moderate in CTB of human placental villi during the first trimester. (c) CD82 was highly expressed in decidual cells (DC). (e) CD82 was highly expressed in the maternal decidua, not detected in EVT and very faint in anchoring villous during the early second trimester. Boxed region is enlarged on the upper panel. (g) CD82 was absent in villous and very faint in EVT during the late second trimester. (i) CD82 was moderate expressed in cytotrophoblast cells invaded into the maternal decidua and highly expressed in syncytiotrophoblast in the third trimester. (b, f, h, j) Immunohistochemical staining with anti-cytokeratin7 (CK7) as a marker of CTB, TC in the first trimester placental vill, EVT in the maternal decidua; (d) Immunohistochemical staining with anti-vimentin as a maker of decidual cells. (k, l, m, n) negative controls (NEG) on sections in which normal IgG was used in place of primary antibody. CTB: cytotrophoblast; STB: syncytiotrophoblast; TC: trophoblast column; EVT: extravillous trophoblast; MD: maternal decidua, AV: anchoring villous W: weeks of pregnancy; Bar represents 100 µm. (**B**) Expression of CD82 in different trophoblast cell lines determined by semiquantitative RT-PCR and Western blotting, respectively. GAPDH was used as an internal control for RT-PCR or loading control for Western blotting. HTR8/SVneo: a human invasive extravillous trophoblast cell line derived from immortalized first trimester trophoblast; B6Tert: immortalized cytotrophoblast cell line; JEG-3: human choriocarcinoma cell lines. (**C**) Immunofluorescence of CD82 in HTR8/SVneo cell lines. Fluorescence signals specific to CD82 antibody were visualized as green, and the nuclei were shown by DAPI staining (blue).

To examine the expression pattern of CD82 protein in human placental villous at different stages of pregnancy, immunohistochemistry was firstly carried out. In first trimester (5 w), the CD82 protein was intensely stained at cytotrophoblasts and trophoblast column (TC) (Figure 1A, a). In the maternal compartment of placenta, staining was seen in decidualized stromal cells in first trimester (7 w) and early second trimester (15 w) (Figure 1A, c, e). But there was no staining in invading interstitial EVTs and the positive staining was very faint in syncytiotrophoblast of anchoring villous (Figure 1A, e). During the late second trimester (25 w), CD82 was not detected in villous and weakly expressed in EVTs (Figure 1A, g). In third trimester (39 w), CD82 was moderately expressed in cytotrophoblast cells that invaded into the maternal decidua and was highly expressed in syncytiotrophoblast (Figure 1A, i). These data showed that CD82 is dynamically expressed in trophoblast lineages throughout gestation. The identification of trophoblast cells and stroma cells were confirmed cytokeratin 7 (CK7) and vimentin staining respectively on a separate adjacent section (Figure 1A, b, d, f, h, j).

Expression of CD82 in several human trophoblast cell lines was also examined by RT-PCR, Western blotting and immunofluorescence microscopy. CD82 mRNA could be detected in HTR8/SVneo and B6Tert (normal trophoblast cell lines), but was very low in JEG-3 cells (human choriocarcinoma cell lines) (Fig. 1B). Similarly, CD82 proteins were present in HTR8/SVneo cells and B6 tert cells, but were also low in JEG-3 cells (Figure 1B). Immunofluorescence microscopy confirmed and further revealed that CD82 proteins were present in HTR8/SVneo cells (Figure 1C).

### CD82 siRNA Promoted EVTs Migration of Villous Explants

**Figure 2:**
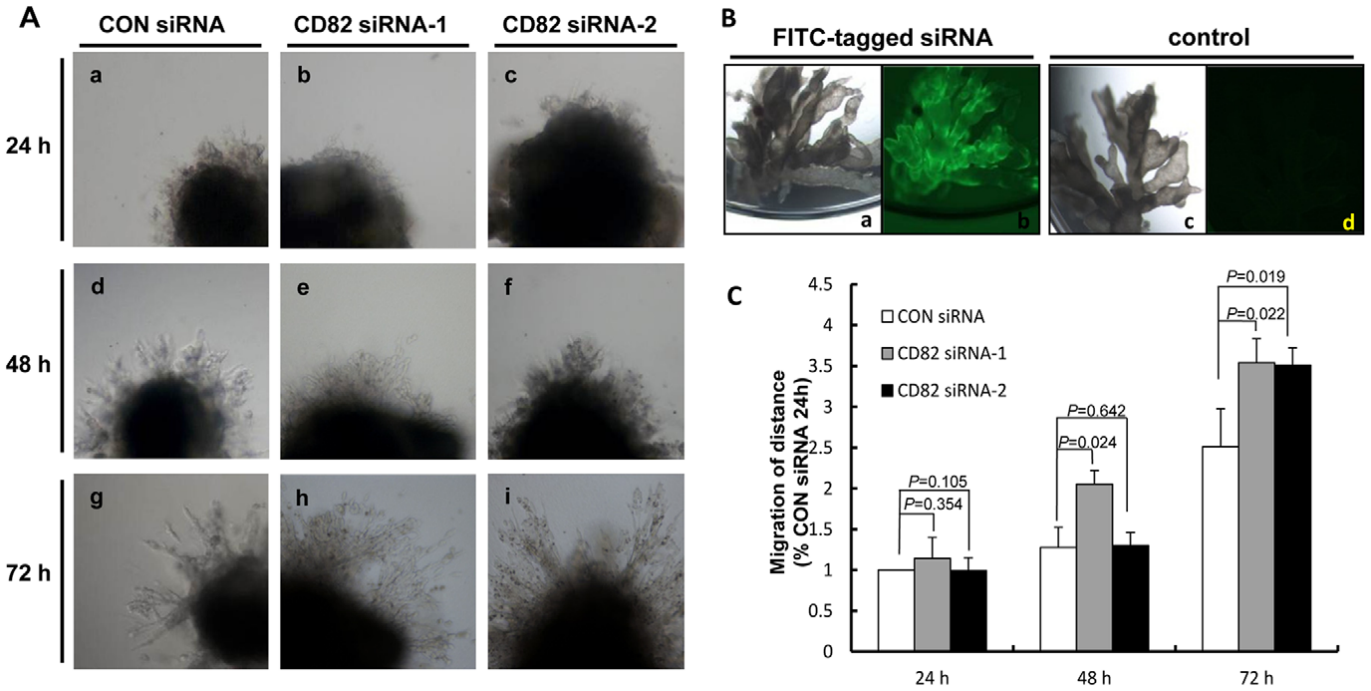
Silencing of CD82 promote trophoblast outgrowth and migration in villous explant cultures. (**A**) Villous explants from 5 to 8 weeks of gestation were maintained in culture for 5 days on matrigel under low (3% O_2_) oxygen tension. Villous explants of CD82 siRNA significantly increase budding and outgrowth of EVT from the distal end of the villous tips comparing with negative control. Serial pictures of villous explants were taken under the light microscope after 24, 48, and 72 h of culture *in vitro.* (**B**) Villi (a) transfected with FITC-tagged siRNA, showing the transfection efficiency (b). Villi (c) as a control transfected nothing, showing the villi background fluorescence (d). (**C**) Three experiments as in **B** were quantified by measuring the the migration distance of villous tip relative to CON siRNA transfected for 24 h. (*t*-test).

Given the evident expression of CD82 on TCs in first trimester placentas, we next investigated whether CD82 have effects on outgrowth of villous explants on Matrigel. EVT cells migrated from the cell column tips and infiltrated the matrix between day1 and day3. Explant culture media contained 500 nM stealth siRNA targeting CD82 and the outgrowth distance of EVTs migration at the Matrigel surface was measured at 24, 48, and 72 h. At 24 h of culture, villous explants anchored on Matrigel and outgrowth took place, but no significant difference was observed between control siRNA and CD82 siRNA-1,-2 (*P>0.05*) (Figure 2A, a, b, c). At 48 and 72 h of *in vitro* culture, CD82 siRNA treated explants displayed a significant increase in the distance of migration compared with the control siRNA (48 h, *P*<0.01;72 h *P*<0.01) (Figure 2A, d and e; g, h and i). The successful introduction of siRNA into the cells of the explant was determined by transfection with a fluorescent-labeled siRNA which exhibiting universal green fluorescence (Figure 2B, b).

### Knocking-Down CD82 by siRNA Promoted Migration and Invasion of HTR8/SVneo Cells

**Figure 3:**
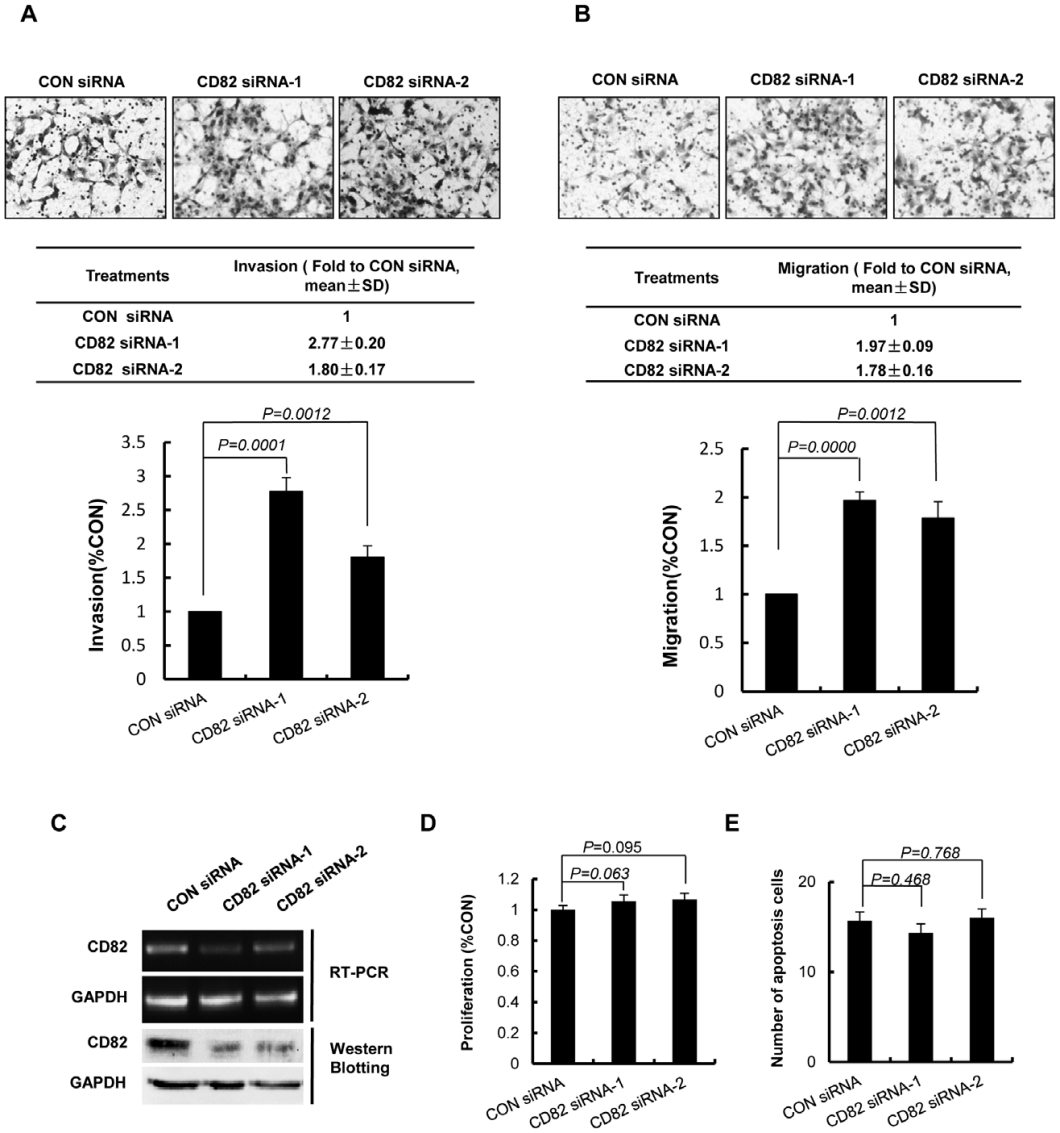
Silencing of CD82 promoted invasion and migration of HTR8/SVneo cells. (**A, B**) Representative images of filters containing invaded cells in Matrigel invasion assay and transwell cell migration assay are shown. The statistical bar graphs show the summary of three independent experiments. (*t*-test) (**C**) Confirmation of RNA interference of CD82 was shown by RT-PCR and Western blotting. GAPDH was used as an internal control in RT-PCR and a loading control in Western blotting. (**D**) MTT assay showed no significant difference on proliferation. (*t*-test) (**E**) CD82 siRNA-1,-2 had no significant effect on apoptosis of HTR8/SVneo cells. (*t*-test).

The evident expression of CD82 in trophoblast cell lines suggested a role in controlling trophoblast cell migration and invasion. Therefore, we further used transwell matrigel invasion assay and migration assay to test the effects of CD82 siRNA on HTR8/SVneo cells from first trimester trphoblast cells. siRNA targeted to CD82 transfected into HTR8/SVneo cells significantly reduced CD82 expression, the knocking down efficiency were examined by RT-PCR and western blotting (Figure 3C). The results showed that HTR8/SVneo cells transfected with CD82 siRNA-1,-2 significantly increased the invasion and migration ability as compared with control siRNA transfected cells. (*P*<0.01) (Figure 3A, B).

### Over-Expression of CD82 Inhibited Migration and Invasion of HTR8/SVneo Cells

**Figure 4:**
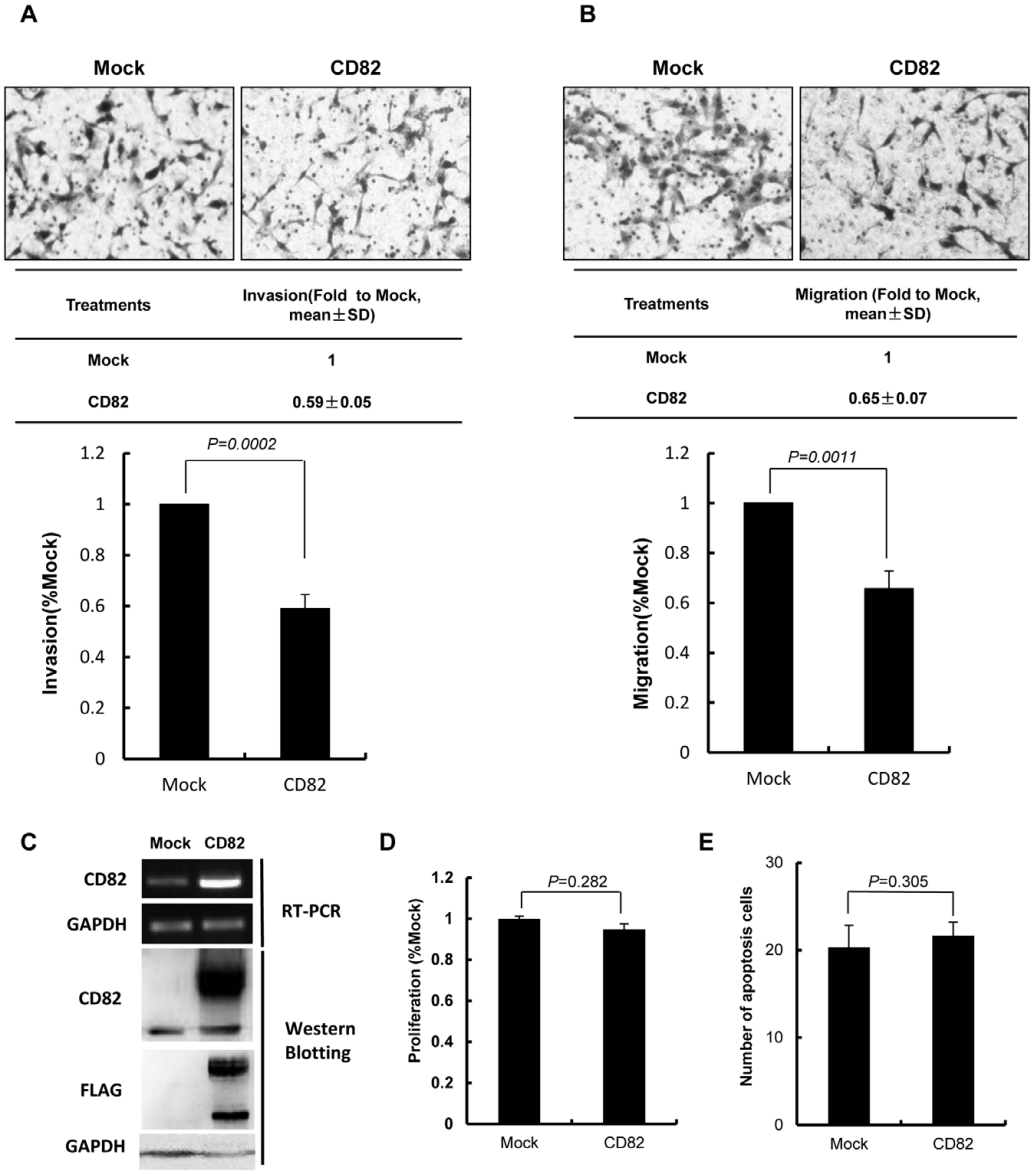
Over-expression of CD82 inhibited invasion and migration of HTR8/SVneo cells. (**A, B**) Representative images of filters containing invaded cells in Matrigel invasion assay and transwell cell migration assay are shown. The statistical bar graphs show the summary of three independent experiments. (*t*-test) (**C**) Confirmation of over-expression of CD82 was shown by RT-PCR and Western blotting. GAPDH was used as an internal control in RT-PCR and a loading control in Western blotting. (**D**) MTT assay showed no significant difference on proliferation. (*t*-test) (**E**) Overexpression CD82 had no significant effect on apoptosis of HTR8/SVneo cells. (*t*-test) Mock: pFLAG-CMV4 empty vector, CD82: pFLAG-CMV4-CD82 vector.

To further explore the function of CD82, we over expressed FLAG-tagged CD82 in the HTR8/SVneo cells. To confirm that the exogenous protein was expressed properly, we performed immunocytochemistry using anti-FLAG antibody and observed that the fusion construct of CD82 was localized to the plasma membrane (Figure S1). Further more, to make sure the antibody targeting CD82 is specific, we employed knocking down, over expression, and immunoprecipitation assay(s) with anti-CD82 and/or anti-FLAG antibodies. (Figure S2). Interestingly, after CD82 over-expression, an additional band at ∼55 KD appeared (Figure S2, A); we believed that is another form due to different level of glycosylation. We then examined the effects of exogenous CD82 on trophoblast cell invasion and migration. As shown in figure 4C, the exogenous expression of CD82 was examined by RT-PCR and western blotting. The results showed that HTR8/SVneo cells overexpressed with CD82 decreased migration and invasion abilities as compared with control groups. (*P*<0.05) (Figure 4A, B).

### Up or Down Regulation of CD82 Shows No Obvious Effects on HTR8/SVneo Cells Proliferation and Apoptosis

To rule out the possibility that the observed invasion/migration changes were due to effects of CD82 on cell proliferation and/or viability, we further investigated the effects of CD82 on trophoblast cell proliferation and apoptosis. HTR8/SVneo cells transfected with siRNA, or pFLAG-CMV4 plasmids encoding CD82, were subjected to MTT assay. The effects of CD82 on trophoblast cell apoptosis were investigated by hoechst 33258 staining. The results showed that either CD82 knock down or over expression has no obvious effects on the cells proliferation and apoptosis compared with control groups. (*P*>0.05, Figure 3D, E; Figure 4D, E).

### Up or Down Regulation of CD82 is Associating with Altered MMP9 Activity and Expression of TIMP1 and 2

**Figure 5:**
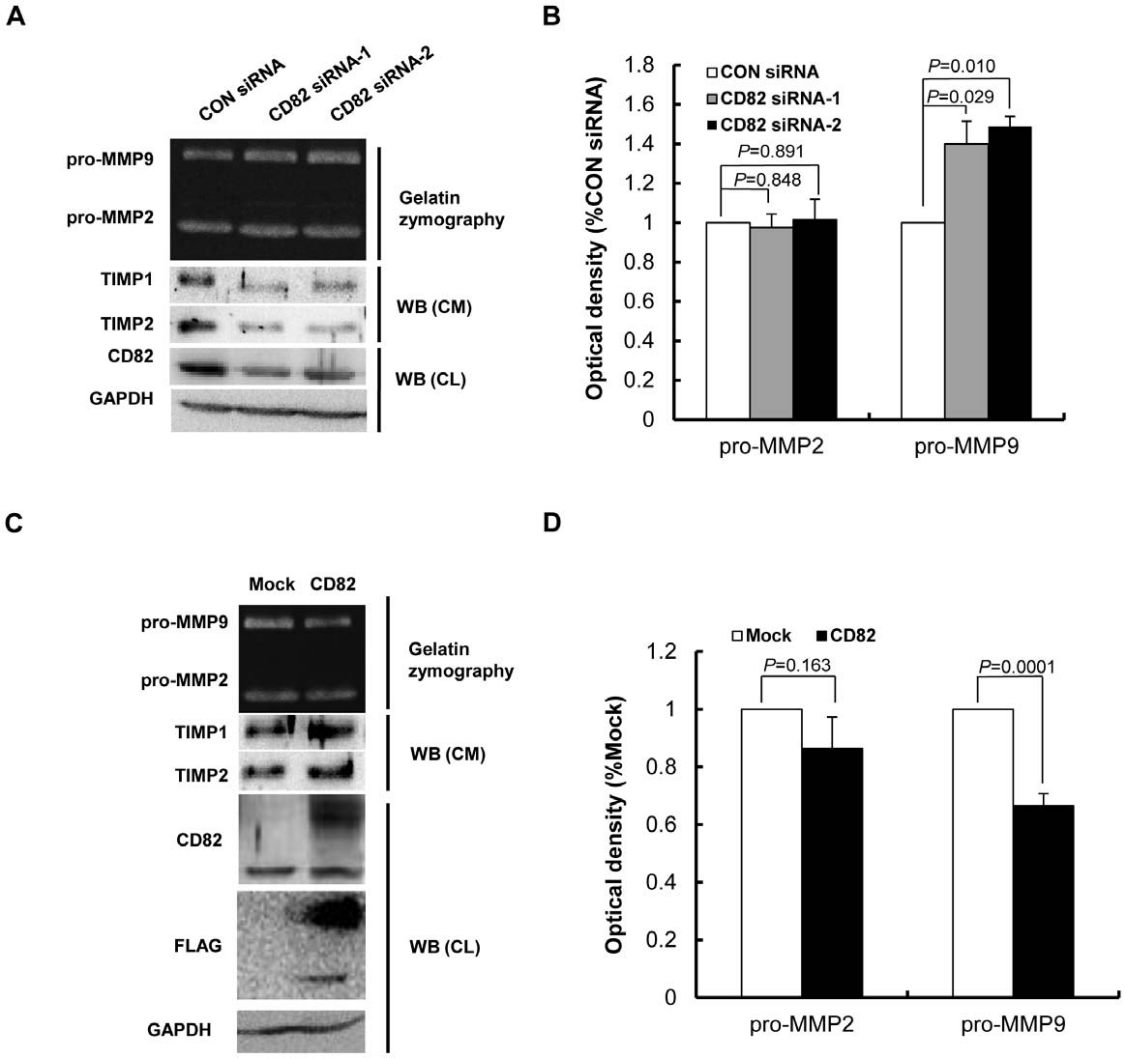
The effect of CD82 on the activities of MMP2, MMP9 and expression of TIMP1, TIMP2. (**A, C**) HTR8/SVneo cells were transfected with CD82 siRNA-1,-2 or pFLAG-CMV4-CD82 plasmid. Total proteins were extracted and western blotting (WB) was performed to detect expression of CD82. Serum-free culture medium was collected for gelatin zymography assay and for Western blotting assay. CD82 siRNA decreased, while over-expression CD82 increased protein levels of both TIMP1 and TIMP2. (**B, D**) Statistical assay of the zymographic results in A or C. CD82 siRNA increased, while over-expressed CD82 decreased the activity of pro-MMP9. Activity of pro-MMP2 was not significantly affected.

Proteolysis played a crucial role in regulation of cell motility. Gelatinases (MMP2 and MMP9) have been implicated in remodeling of extracellular matrix in trophoblast invasion process. To investigate the effects of CD82 on proteolysis in mediating trophoblast invasion, MMP2 and MMP9, and their inhibitors TIMP1 and TIMP2, were examined. Gelatin zymography was performed to detect the gelatinase MMP2 and MMP9 activity in HTR8/SVneo cells transfected with CD82 siRNA or overexpression of CD82. The results revealed that the expression level of CD82 is negatively related to pro-MMP9 activities and positively related to the expression level of TIMP1 and 2 (*P*<0.01) (Figure 5A, C,). However, the level of pro-MMP2 shows no significant changes. (*P*>0.05) (Figure 5A, C,).

## Discussion

In the present study, we provided direct evidence that CD82 is not only expressed in the maternal compartment in the decidua, but also localized in the trophoblast cell lineage and plays an important role in inhibiting trophoblast outgrowth and invasion. Our data demonstrated that CD82 was strongly expressed in the villous of syncytiotrophoblast, cytotrophoblasts and extravillous trophoblast cells that invasive into maternal decidua, which is consistent with the previous microarray data that CD82 is highly expressed in human placenta [25], [26]. Using well-established in vitro models, we further showed that knocking-down of CD82 resulted in significantly promoted cytotrophoblasts outgrowth and EVT migration through Matrigel, which was associated with the fact that CD82 suppressed trophoblast invasion and gelatinolytic activity. Moreover, these findings were consistent when using a well-defined trophoblast cell line HTR8/SVneo. Our data demonstrated that CD82 may play an important role in trophoblast invasion without affecting trophoblast viability/proliferation, possibly by regulating invasion associated proteases in direct or indirect ways during early pregnancy.

The metastasis suppression role of CD82 is well-defined in hepatocarcinoma, melanoma, sarcoma, pancreatic and breast cancer cell lines [27]–[31], and it has been observed that down regulation of CD82 mRNA and protein is associated with advanced stages of many malignancies including prostate, colon, lung, pancreatic, breast, ovarian and other cancers, suggesting the loss of CD82 contribute to the increased invasiveness of these cancers. There are striking similarities between trophoblast cells and tumor cells with regard to proliferative and invasive properties [32], and the physiological balance of trophoblast invasiveness is regulated by cross-talk between trophoblasts and DSCs in a paracrine and autocrine manner, which involves growth factors, adhesion molecules, cytokines and chemokines [10], [11]. The potential role of CD82 at maternal-fetal interface in regulating trophoblast invasion has been studied by other groups [21]. It is reported that the expression of CD82 in decidual cells at the human maternal-fetal interface is important in controlling over-invasion of trophoblast cells in a paracrine manner [21]–[24]. However, whether CD82 is also expressed in trophoblast lineages at different stage of gestation remains unclear. Through data mining from existing microarray database, we interestingly found that CD82 was strongly expressed in the human placenta, suggesting a possible contribution from the trophoblast lineage. Moreover, within our ability to detect two well-established trophoblast cell lines, HTR8/SVneo and B6 tert, we could constantly detect strong expression of CD82 at transcription level, while using two human choriocarcinoma cell lines JEG-3 and JAR, the CD82 expression is very low or could not be detected (similarly, an independent group has also shown negative expression of CD82 in another trophoblast cancer cell line BeWo). These clues raised the possibility that CD82 might be important in normal trophoblast cell (cell lines) function while trophoblast cancer cell (cell lines) has lost CD82 expression. Therefore, in this study, we further managed to confirm the CD82 expression in trophoblast cells at both transcription and protein level. Here we used commercial antibodies from several companies (Abcam, Santa Cruz, abgent) to examined expression of CD82 at maternal-fetal interface obtained from first trimester. In contrary to some of previously published data, 3 out of 4 antibodies (SC-5540/ab66400/AP6250a) used in our system could detect obvious expression of CD82 at both maternal decidua and trophoblast cells, and could also observe evident staining in normal trophoblast cell lines. These data led us to believe the existence of CD82 in trophoblastic lineages. Our functional studies by knocking down and overexpression of CD82 in explant culture and trophoblast cell line further provided direct evidences to support the role of CD82 in trophoblast function.

As for the discrepancies obtained compared with other groups reporting negative expression of CD82 in trophoblast, one possibility lies in the different timing of sample collection. Since CD82 expression might have dynamic level and patterns at different trimesters, it is possible that while in the first trimester when trophoblast is highly invasive, CD82 was probably required to inhibit excessive invasion, thus easily to detect. Also, samples from different individuals under different genetic backgrounds might contribute to the observed expression variations. Most possibly and intriguingly, we’ve now known that human CD82 gene showed very diverse splice forms (14 splice forms according to ensemble database and 10 of them could be transcribed into different forms of proteins: http://www.ensembl.org/Homo_sapiens/Gene/Summary?g=ENSG00000085117r=11∶44585977-44641913). Moreover, it has been demonstrated that CD82 has more than one splice forms as demonstrated previously [17]. As each commercial antibodies only recognize one small epitome of the CD82 protein, there is a reasonable possibility that the expression discrepancies revealed by different antibodies might imply that there are dominant expression of different isoforms of CD82 at trophoblast and decidual compartments of maternal-fetal interface, which is an interesting issue warrants further detailed investigations.

As for the downstream signaling and mechanism that responsible for inhibition of CD82 in trophoblast invasion at maternal-fetal context, it is well-established that trophoblast invasion are closely correlative with the expression of MMPs [33], [34], which are capable of degrading extracellular matrix to facilitate the invasion process. This led us to look into the effect of CD82 on MMPs activities. Among members of MMP family, MMP2 and MMP9 were most extensively studied and was demonstrated to play major roles in pathways of trophoblast invasion. Indeed, it has been previously reported that CD82 suppresses tumor invasion by MMP9 inactivation via TIMP1 up-regulation in carcinoma cell line [35]. Here our data indicated that the trophoblast cells shares similar mechanisms in control regulated-trophoblast invasion.

In summary, the present data demonstrated that CD82 is expressed in trophoblast lineages at maternal-fetal interface of first trimester. CD82 restricts invasion of trophoblast cells, which is associated with decreased gelatinases. CD82 might be an important participator in maintaining normal placenta function and its pathophysiological role in human pregnancy awaits further investigations.

## Materials and Methods

### Tissues Collection

Human placental tissues from 5 to 9 weeks (indicated as the first trimester) were obtained from healthy women undergoing legal abortion for nonmedical reasons; term placentas were collected after vaginal delivery or caesarean birth. Ethical approval was granted by Ethic Committee of the Xuan Wu Hospital in Beijing. All women were informed and signed consent to donate placenta for scientific research use. This study and the use of samples were under standard experimental protocols approved by the Ethics Committee of the Chongqing Medical University. All the tissue samples were collected and stored in ice-cold DMEM (Invitrogen), transported to the laboratory within 30 minutes after surgery, and washed with ice-cold PBS before fixation(first trimester = 5, normal third trimester = 5).

### Cell Culture

The human extravillous trophoblast cell line HTR8/SVneo was widely used as a model for the first trimester EVT invasion and migration. HTR8/SVneo cells used in this study were from Professor Benjamin K Tsang (Department of Obstetrics & Gynecology and Cellular & Molecular Medicine, University of Ottawa; Chronic Disease Program, Ottawa Health Research Institute, Ottawa, ON K1Y 4E9, Canada), as previously mentioned [36]. Cells were cultured in RPMI 1640(Gibco, MA) media containing 10% fetal bovine serum (Hyclone, UT), 100IU/ml penicillin and 100 µg/ml streptomycin, and incubated under 5% CO_2_ at 37°C. Cells cultured in serum free media for concentrating supernatants after siRNA transfection.

### Semiquantitative RT-PCR

Total RNA was extracted from HTR8/SVneo cells using TRIzol regent (Invitrogen, CA) and purified as described in the manufacturer’s instruction. Briefly, after homogenization, chloroform was added and mixed thoroughly, incubated for 3 minutes at room temperature and centrifuged for 15 min (12,000 g, 4°C). The aqueous phase containing the RNA was removed and added to isopropanol, incubated for 10 minutes at room temperature and centrifuged for 10 min (12,000 g, 4°C). The supernatant was removed and the RNA pellet washed in 75% ethanol and centrifuged for 5 min (7,500 g, 4°C). The supernatant was removed and the RNA resuspended in DEPC-treated water. 2 µg of RNA were reverse transcribed in a 20 µl reaction containing Superscript II reverse transcriptase (Invitrogen, CA). The PCR was conducted in a total volume of 25 µl at different cycles ranging from 22–30 cycles, determined by PCR amplification of the target genes. The GAPDH was employed as an internal control. PCR proceed for 22–30 cycles (30 sec denaturation at 95°C, 30 sec annealing at 58°C, and 30 sec elongation at 72°C) after an initial 5 min denaturation step at 95°C. The primers used in this study include CD82 (Forward: 5′-CTGGGGCTGTACTTTGCTTTC-3′, Reverse: 5′-CAGAAGCCCTTCCTCACAGAA-3′) and GAPDH (Forward: 5′-AGCCACATCGCTCAGACA-3′, Reverse: 5′-TGGACTCCACGACGTACT-3′) CD82 full-length (Forward: 5′-CCTGCTGCTGTGTGGACGA-3′, Reverse: 5′-GCACTGGTTTCGTGGAAGGA-3′). Amplifications were analyzed by electrophoresis on agarose gels containing ethidium bromide.

### Western Blotting

Cells were lysed directly in SDS gel loading buffer (60 mM Tris–HCl, pH 6.8, 2% SDS, 10% glycerol, 5% beta-mercaptoethanol, 0.001% bromphenol blue). Culture media were concentrated using Microcon YM-3 centrifugal filter (Millipore Corp. Bedford). Equal amount of denatured protein per well were subjected to SDS-PAGE according to standard protocols. Separated proteins were transferred electrophoretically onto a nitrocellulose membrane (Pall Corporation, Pensacola, FL). After being blocked with 5% skim milk in TBST for 1 h at room temperature, the membrane was sequentially incubated with primary antibodies against CD82 (ab66400, Abcam, Cambridge, UK Inc.),TIMP1(SC-5538, Santa Cruz, CA), TIMP2 (SC-5539, Santa Cruz, CA), FLAG (F1804, Sigma-Aldrich, Inc., St. Louis) and GAPDH (ab37187, Abcam, Cambridge, UK) overnight at 4°C, and washed three times for 10 min per wash with TBST. A subsequent incubation with monoclonal HRP-conjugated antibody was carried out for 1h at room temperature in 5% skim milk in TBST, and three times washes with TBST were performed. Immunoreactive bands were detected using enhanced chemiluminescence (Pierce Chemical Co., Rockford, IL).

### Immunohistochemistry

Immunohistochemistry was performed on formalin-fixed, paraffin-embedded 5 µm sections using the biotin-streptavidin-peroxidase (SP) and diaminobenzidine (DAB, Zhongshan Golden Bridge Crop., Beijing, China) kit, as previously reported [21]. Sections were dewaxed in xylene and rehydrated through gradient of ethanol. Slides were microwaved in citrate buffer (10 mM citrate sodium, 10 mM citric acid, pH 6.0) or Tris-EDTA buffer (10 mM Tris, 1mM EDTA, pH 9.0) for 15 min to retrieve antigen, and then sequentially incubated with 3% hydrogen peroxide in water for 10 min to quench endogenous peroxidase activity.

After blocking with normal goat serum for 1 hour, sections were incubated with primary antibodies against CD82(SC-5540 Santa Cruz, CA) at 4 ug/ml, vimentin (ab8069, Abcam, Cambridge, UK) and cytokeratin(CK)7 (ab20206, Abcam) at 5 ug/ml overnight at 4°C. After washing with PBS, the sections were incubated with biotinylated secondary antibody followed by streptavidin-peroxidase, and was detected with DAB solution. Negative control was performed by replacing the primary antibody with normal goat serum or IgG from the host species of the primary antibody.

### Immunofluorescence

HTR8/SVneo cells or 293FT cells (transfected with FLAG-tagged CD82 plasmid) were fixed in 4% paraformaldehyde/PBS for 20 min at room temperature. After fixation, cells were washed in PBS three times for 15 min and permeabilized with 0.1% Triton/PBS for 10 min. Cells were incubated in 1% BSA/PBS for 1 hour at room temperature. Then, cells were incubated with anti-CD82 antibody(ab66400, Abcam) or anti-FLAG(F1804, sigma) antibody overnight at 4°C followed by fluorescein isothiocyanate (FITC)-conjugated anti-rabbit or anti-mouse IgG (Zhongsan Golden Bridge Crop., Beijing, China)at a 1∶100 dilution for 1 hour at room temperature. Nuclei were stained with 4′, 6′-Diamidino-2-phenylindole (DAPI) for 3 min. Finally, cells were viewed under a laser-scanning confocal microscope (Zeiss).

### Explant Culture

The explant culture was performed as described previously [13]. In brief, small pieces of tissue (2–3 mm) from tips of first trimester placental villi (5 w–8 w) were dissected. The samples of dissected villi were explanted in Millicell-CM culture dish inserts (0.4 µm pore size, Millipore, Carrigtwohill, Co. Cork, Ireland) precoated with phenol red-free matrigel substrate (Becton Dickinson, Bedford, MA). Inserts were placed into 24-well culture dishes (Costar, Cambridge, MA). The explants were cultured in serum-free F12/DMEM media with 500 nM siRNA, 100IU/ml penicillin and 100 µg/ml streptomycin, at 3% O_2_/5%CO_2_/92%N_2_. EVT sprouting and migration from the distal end of the villous tips were recorded daily for up to 4 days. The extent of migration (*i.e.* the distance from the cell column base to the tip of the outgrowth) was measured at defined positions with SPOT Advanced software. All explants experiments with cultured villi were repeated three times and were replicated in four separate sets of explants.

### RNA Interference (RNAi) and Over-Expression of CD82

HTR8/SVneo cells were transfected with 100 nM CD82 siRNA-1, 2 (1. 5′-UAUUUGGUGACUUUGAUACAGGCUG-3′, 2. 5′-AUACAAUAUACACACCCUUGAGGGC-3′ Invitrogen, MD; Genbank ID for CD82: NM_002231.3), control siRNA(a universal negative control, Invitrogen, MD) with Lipofectamine™ 2000(Invitrogen, MD)as recommended by manufacturer. The transfection efficiency was more than 90% by using fluorescent-labeled siRNA.

The full-length CD82 was subcloned into pFLAG-CMV4 vector, and 6 µg were transfected into HTR8/SVneo cells at 70% confluence for 60mm dish. The control was instead with pFLAG-CMV4 empty vector. The transfection efficiency was about 40% by counting FLAG positive cells using immunocytochemistry (Figure 1S, A).

### Matrigel Invasion Assay

Invasion assay was performed in Matrigel (BD, MA)-coated transwell inserts (6.5 mm, Costar, Cambridge, UK) containing polycarbonate filters with 8 µm pores size, as described previously [37]. Briefly, the inserts were pre-coated with 100 µl of 1 mg/ml Matrigel matrix at 37°C for 4 h for gelling. 1×10^5^ HTR8/SVneo cells in 200 µl serum-free medium were plated in the upper chamber, whereas medium with 10% fetal bovine serum was added to the lower well. After incubating for 24 h, the cells on the Matrigel side of the insert were scraped by cotton swab. The inserts were then fixed in methanol for 10 min at room temperature and stained with haematoxylin and eosin (Zhongshan Golden Bridge Corp, Beijing, China). Cells invaded to the other side of the insert were counted under a light microscope (Olympus IX51, Japan) in ten random fields at a magnification of ×200. The assay was repeated three times, and the results are represented as means of invasion percentage (%) ±SD in cell invasion compared with control. Conditional culture medium was collected for gelatinolytic activity assay.

### Transwell migration assay

The migratory ability of HTR8/SVneo cells was determined by their ability to cross the 8 µm pores of migration chambers. Methods used in cell migration assay were similar to Matrigel invasion assay except that the transwell insert was not coated with Matrigel.

### MTT Assay

After tranfection of siRNA, HTR8/SVneo cells were subjected to invasion and migration assay, and the remaining cells were utilized for MTT assay to measure cell proliferation. HTR8/Svneo cells were seeded at 0.5×10^4^/well in 96-well. The culture medium was changed after 20 h to 100 µl MTT reagent (3-[4, 5-dimethylthiazol-2-yl]-2, 5-diphenyltetrazolium bromide; Apllygen Corp. Beijing, China). The MTT reagent was gently removed 4 h later and 100 µl DMSO was added in each well. The optical density of each well was measured at 570 nm wavelengths (Beckman DU530, Fullerton, CA). The experiment was performed in triplicates.

### Hoechst 33258 Staining

Hoechst 33258 staining of HTR8/SVneo cells was performed to evaluate the cell death pattern after treatments of control siRNA and CD82 siRNA. Twenty-five microliter of cell suspension (about 0.5×10^4^ cells) was incubated with 33258 (Sigma-Aldrich, Inc., St. Louis) solution. Cell suspension was placed onto a microscopic slide covered by a coverslip. The number of apoptotic cells in 200 total cells was counted under a fluorescence microscope microscope (Olympus IX51, Japan) in ten random fields at a magnification of ×200.

### Gelatin Zymography

Analysis of gelatinolytic activity was performed using 10% (w:v) polyacrylamide gel s containing 0.5 mg/ml gelatin (Difco Laboratories, Detroit, MI) as previously reported [33]. Briefly, conditioned medium was diluted in 4X non-reducing sample buffer (8% SDS (w:v), 0.04% bromophenol blue (w:v), 0.25 M Tris·HCl pH 6.8] and incubated at 37°C for 30 min, and equal amounts of protein were subjected to substrate-gel electrophoresis. After electrophoresis, the gel was washed twice in 2.5% Triton X-100(v:v) in 50 mM Tris–HCl (pH 7.5) for 30 min at room temperature to remove SDS, and then incubated in calcium assay buffer (50 mM Tris, 10 mM CaCl_2_, 1 mM ZnCl_2_, 1% Triton×−100, pH 7.5) for 24 h at 37°C. Gels were stained with Coomassie Brilliant Blue R250 in 50% methanol and 10% acetic acid and destained in 10% acetic acid to reveal zones of gelatinase activity.

### Statistical Analysis

Each experiment was performed in triplicates. The bands from RT-PCR, Western blotting and gelatin zymography were quantified by MetaView Image Analyzing System (Version 4.50; Universal Imaging Corp., Downingtown, PA). Results were presented as means±SD. Statistical analyses include one-way ANOVA and *t*-test which were performed using the Statistical Package for Social Science Science (SPSS for Windows package release 10.0; SPSS Inc., Chicago, IL) and indicated in Results and figure legends. *P*<0.05 was considered as statistically significant.

## Supporting Information

Figure S1 **Transfection efficiency and expression pattern of the pFLAG-CMV4-CD82 plasmid in HTR8/SVneo and 293FT cell lines.** (**A**) Immunocytochemistry was performed by using Flag antibody (visualized as green signal) in CD82 over-expressed HTR8/SVneo cells. The nuclei were shown by DAPI staining (blue). The green signals indicate that the efficiency of transfection is more than 30%, and FLAG-CD82 fusion protein localizes to the plasma membrane properly. (**B**) We use 293FT cells to further confirm the results. Bar represents 100 µm.
(TIF)

Figure S2 **CD82 antibody evaluation/validation** (**A**) CD82 knockdown and over-expression assay in HTR8/SVneo cells. Antibody anti-CD82 was used. The arrow indicates two specific bands in over-expression assay. GAPDH were used as loading control. Line 1: HTR8/SVneo cells transfected with control siRNA Line 2–4: HTR8/SVneo cells transfected with CD82 siRNA-1,-2,-3. Line 5: HTR8/SVneo cells with transfection reagent only Line 6: HTR8/SVneo cells transfected with 3 µg pFLAG-CMV4 empty vector Line 7: HTR8/SVneo cells transfected with 3 µg pFLAG-CMV4-CD82 plasmid Line 8: HTR8/SVneo cells transfected with 2 µg pFLAG-CMV4 empty vector Line 9: HTR8/SVneo cells transfected with 2 µg pFLAG-CMV4-CD82 plasmid (**B**) CD82 over-expressed HTR8/SVneo cells, whole cell lysates and proteins immunoprecipitated using FLAG-beads. Line 1, 4: Whole cell lysates of CD82 over-expressed HTR8/SVneo cells Line 2, 3: Immunoprecipitated proteins using Flag beads from CD82 over-expressed HTR8/SVneo cell lysates.
(TIF)
