# Supplementary figures and images for: Expression of CD82 in Human Trophoblast and Its Role in Trophoblast Invasion
Source: PLoS One. 2012 Jun 5;7(6):e38487. doi: 10.1371/journal.pone.0038487 (PMC3367946; doi:10.1371/journal.pone.0038487)

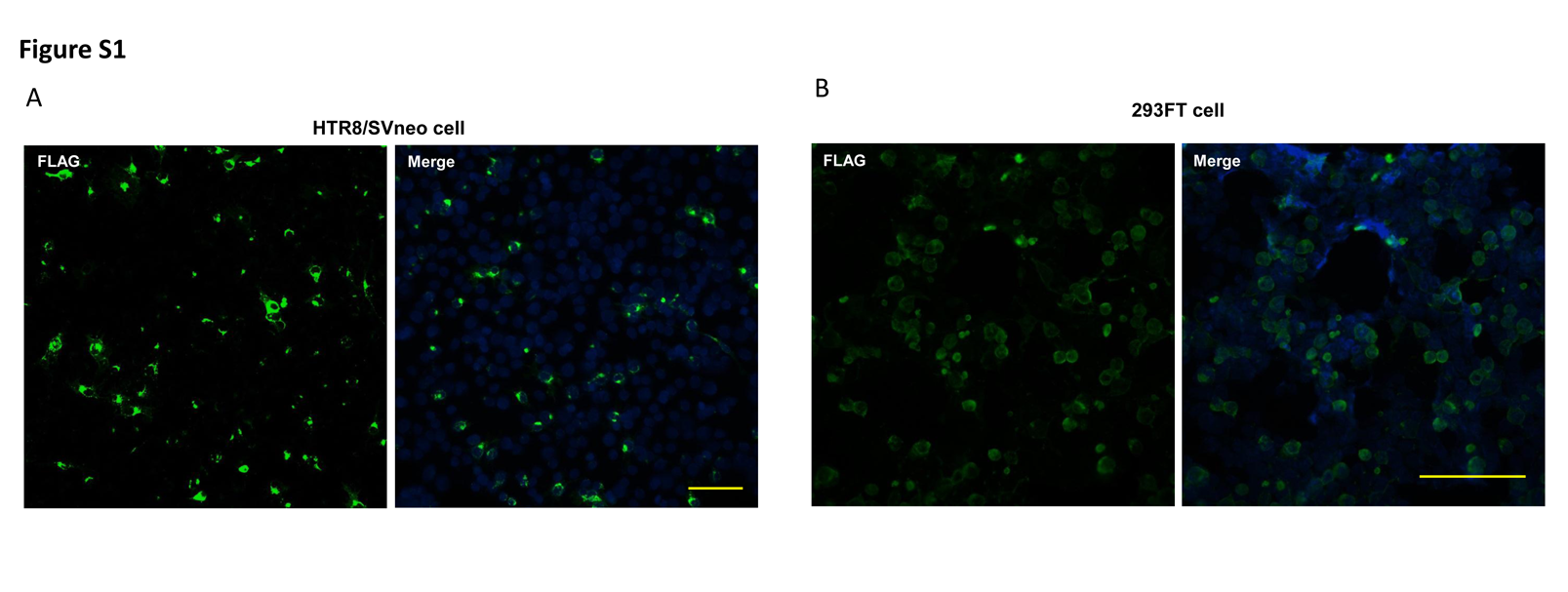

Supplement: Figure S1 — Transfection efficiency and expression pattern of the pFLAG-CMV4-CD82 plasmid in HTR8/SVneo and 293FT cell lines. (A) Immunocytochemistry was performed by using Flag antibody (visualized as green signal) in CD82 over-expressed HTR8/SVneo cells. The nuclei were shown by DAPI staining (blue). The green signals indicate that the efficiency of transfection is more than 30%, and FLAG-CD82 fusion protein localizes to the plasma membrane properly. (B) We use 293FT cells to further confirm the results. Bar represents 100 µm. (TIF) [file pone.0038487.s001.tif]

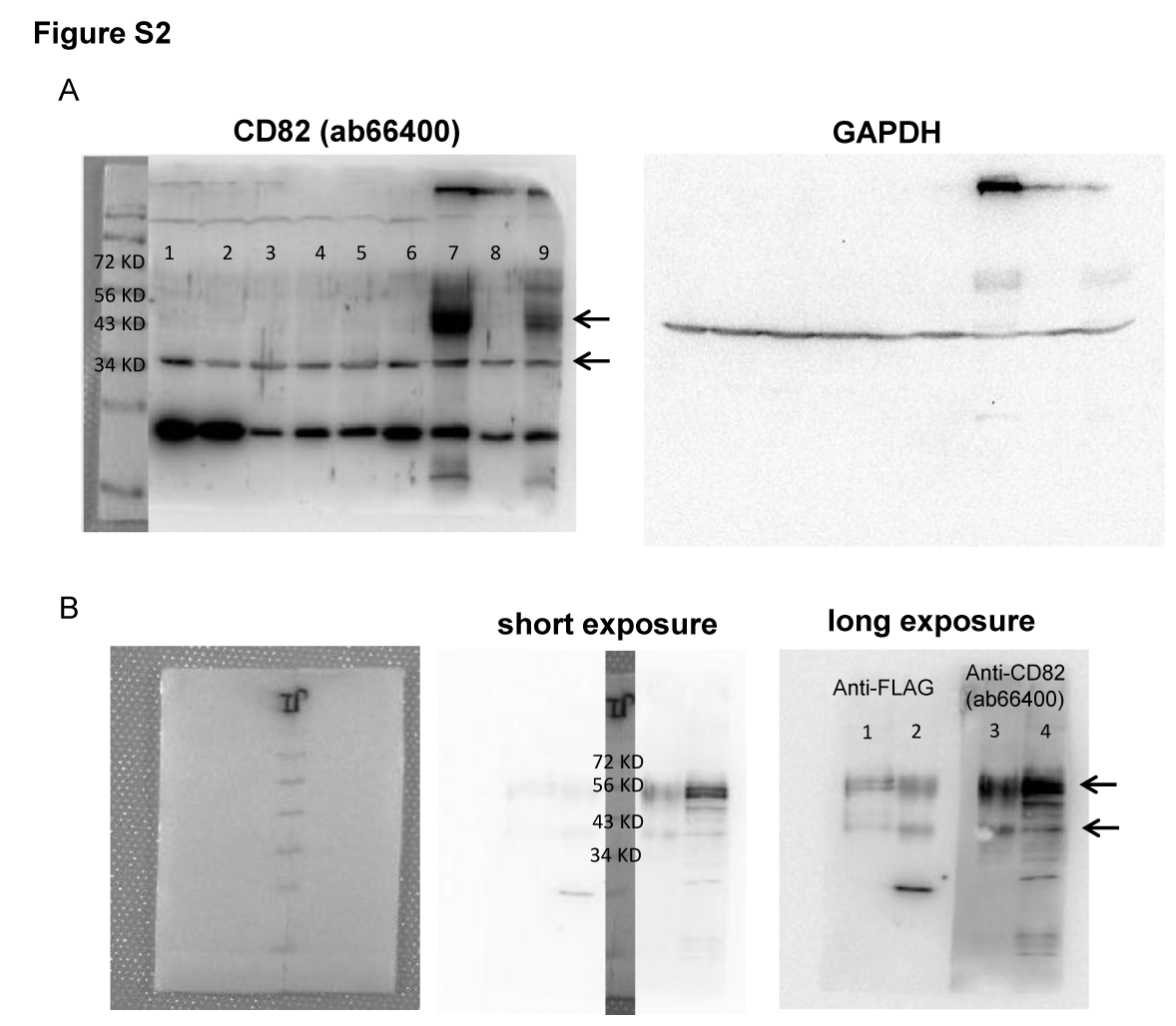

Supplement: Figure S2 — CD82 antibody evaluation/validation (A) CD82 knockdown and over-expression assay in HTR8/SVneo cells. Antibody anti-CD82 was used. The arrow indicates two specific bands in over-expression assay. GAPDH were used as loading control. Line 1: HTR8/SVneo cells transfected with control siRNA Line 2–4: HTR8/SVneo cells transfected with CD82 siRNA-1,-2,-3. Line 5: HTR8/SVneo cells with transfection reagent only Line 6: HTR8/SVneo cells transfected with 3 µg pFLAG-CMV4 empty vector Line 7: HTR8/SVneo cells transfected with 3 µg pFLAG-CMV4-CD82 plasmid Line 8: HTR8/SVneo cells transfected with 2 µg pFLAG-CMV4 empty vector Line 9: HTR8/SVneo cells transfected with 2 µg pFLAG-CMV4-CD82 plasmid (B) CD82 over-expressed HTR8/SVneo cells, whole cell lysates and proteins immunoprecipitated using FLAG-beads. Line 1, 4: Whole cell lysates of CD82 over-expressed HTR8/SVneo cells Line 2, 3: Immunoprecipitated proteins using Flag beads from CD82 over-expressed HTR8/SVneo cell lysates. (TIF) [file pone.0038487.s002.tif]
